# 3D Supergravity in the Batalin–Vilkovisky Formalism

**DOI:** 10.1007/s00023-025-01588-6

**Published:** 2025-05-27

**Authors:** A. S. Cattaneo, N. Moshayedi, A. Smailovic Funcasta

**Affiliations:** 1 Institut für Mathematik, Universität Zürich, Winterthurerstrasse 190, CH-8057 Zürich, Switzerland; 2 Department of Physics, ETH Zürich, Otto-Stern-Weg 1, CH-8093 Zürich, Switzerland

## Abstract

Three-dimensional supergravity in the Batalin–Vilkovisky formalism is constructed by showing that the theory including the Rarita–Schwinger term is equivalent to an AKSZ theory.

## Introduction

In this paper, we construct the BV action [4] for supergravity in three dimensions (see e.g. [1, 3, 10, 28] and references therein).

Our strategy is to start considering the minimal coupling of spin $\frac{3}{2}$ fermions to *BF* theory (with Lie algebra $\mathfrak {so}(2,1)$), where the BV action is easy to construct, since we realise this as an AKSZ theory [2].

Three-dimensional *BF* theory is on shell equivalent to gravity in the Palatini–Cartan formalism [31], and the off-shell equivalence in the BV formalism has been constructed in [19]. Similarly, a super version of three-dimensional *BF* theory is on shell equivalent to supergravity in the Palatini–Cartan formalism [1].

The novel contribution of this paper is the extension of the BV transformation of [19] in the presence of spin $\frac{3}{2}$ fermions, see Theorem 4.11. The advantage is that the rather involved BV structure of three-dimensional supergravity (for which we found no explicit expression in the literature) is obtained from the straightforward BV structure of super *BF* theory via the AKSZ formulation. The main point of the transformation is that the AKSZ symmetries are expressed as covariant derivatives of ghosts which are Lie algebra-valued 0-forms, whereas in (super)gravity we want to see ghosts for (super)diffeomorphisms explicitly.

One can also observe that in our approach supergravity is “discovered”, in the sense that the resulting BV operator turns out to contain the local supersymmetry transformations. More precisely, switching to zero all the ghosts but for the fermionic ghost $\varepsilon$, we get

$$\begin{aligned} Qe&= {\overline{\psi }}\, \rho \varepsilon , \\ Q\psi&= D\varepsilon , \end{aligned}$$

where *e* denotes the dreibein, $\psi$ the spin $\frac{3}{2}$ field, $\rho$ the spin representation, and *D* the covariant derivative (note that the spin connection is not affected by this transformation). Moreover, the ghost for diffeomorphisms accordingly transforms with a term $\frac{1}{2} {\overline{\varepsilon }}\, {e^{-1}} (\rho )\varepsilon$, which encodes the fact that two supersymmetry transformations yield a translation.

The BV structure we obtain for supergravity in three dimensions is considerably simpler than in four, where the BV action is known to require nonlinear terms in the antifields (see [5] for the half-shell formulation and [12]for the Palatini–Cartan formulation). One reason for this may be that three-dimensional gravity is topological and remains so when adding spin $\frac{3}{2}$ fermions.

In this note, we focus on the Minkowski signature and on the spin $\frac{3}{2}$ Majorana representation. However, these results are readily generalised to the Euclidean case (or to alternative signatures) and to other real representations of $\mathfrak {spin}(2,1)$ or $\mathfrak {spin}(3)$, respectively.

### Motivation

The Palatini–Cartan–Holst (PCH) formalism was conceived in order to bring Einstein’s general relativity closer to the language of gauge theories,^1^ and based on the work in [17] it was proven in [19] that in three dimensions Palatini–Cartan (PC) gravity is strongly equivalent to a *BF* theory, result that serves as a basis for the current paper. Here we will show that this strong equivalence persists even when we incorporate the Rarita–Schwinger term to the action,^2^ which further allows us to define a Batalin–Vilkovisky (BV) extension of 3D supergravity where both the invariance under spacetime diffeomorphisms and the supersymmetry are explicitly encoded in their corresponding ghost fields.

Such result might serve as the starting point for the analysis of the boundary structure of this theory through the BV–BFV formalism [14] developed by Cattaneo, Mnev and Reshetikhin, hence joining a broader effort to better understand how gravity coupled to different kinds of matter fields should be described, as well as how to quantise such theories, even in the presence of non-trivial boundaries. Recent papers in this line include [6, 9, 13, 18, 20], to name a few. A further development of the results of this paper would be its extension to all lower-dimensional strata, as was done for PC theory in [7], and the construction of its space of quantum states, which for Einstein–Hilbert theory was done in [8], leveraging the relation with PC theory.

Concerning the structure of the document, the second section consists of a short summary of the BV formalism [4] and of the AKSZ procedure [2]. The third section, in turn, presents BV and *BF* gravity and leads to the fourth and last section, where we present supergravity and build its BV extension. Besides these sections, the reader can find appendix where we will compile a series of results of graded geometry that we shall use in the rest of the article. Sources reviewing graded geometry are the review [15] or the books [22, 24]. For an approach to three-dimensional supergravity in the context of graded geometry, see also [10, 11]. Lastly, we let the reader know that this article is an improved adaptation of the content in the master thesis of the third author [29].

### Notation

- $\mathbb {N}$ denotes the set of natural numbers *including* 0.
- $[\![m,n]\!] = \{k\in \mathbb Z\ |\ m \le k \le n\}$ denotes a range of integers.
- Given a Lie group *G*, its associated Lie algebra is denoted by $\mathfrak g$.
- All forms of products are left implicit and deduced from the context, unless some ambiguity is present, e.g. $\lambda \cdot u\otimes v =: \lambda u v$.
- In the absence of parentheses, a derivation *D* acts only on the element directly adjacent to it: $aDbc :=aD(b)c$.
- Einstein’s summation convention for pairs of upper and lower indices is generally assumed: $x^i y^i \ne x^i y_i :=\sum _{i=j} x^i y_j$.
- Indices with a bar on them are not summed over: $x^{\bar{\imath }}y_{\bar{\imath }}\ne x^i y_i$.
- $\delta ^i_j$ denotes Kronecker’s delta: $\delta ^i_j = 1$ for $i=j$, $\delta ^i_j = 0$ otherwise.
- In the context of gravity or special relativity, Greek letters designate spacetime indices while Latin indices designate Lorentz bundle indices.
- The equivalence sign “$\equiv$” is used to designate equality *on shell*.
- There are multiple fields associated with a field $\psi$:
  - ${\psi ^{*}}$ is it complex conjugate,
  - ${\psi ^{\dagger }}$ is its Hermitian conjugate,
  - ${\overline{\psi }}$ is its Dirac conjugate,
  - ${\psi ^{+}}$ is its associated antifield.

## Classical BV formalism

The path integral formalism for quantum field theory relies on the possibility of integrating out the quadratic terms in the Lagrangian density defining the action, which is achieved through a generalisation to field theory of the saddle point method—known as *Feynman–Laplace method*— requiring the critical points of the action to form a finite subset of its support. However, precisely because of the “continuity”—as opposed to “discreteness”—of topological groups, in theories described by a Lagrangian with gauge freedom one can smoothly deform a critical point into another critical point, resulting in critical loci that are themselves submanifolds. This spoils the applicability of the aforementioned method, making manifest the need for machinery that selects discrete subsets of the critical locus of an action. This is precisely the issue that both the BRST and the BV formalisms address.

We choose to employ the latter because it has a greater range of applicability than the former and also because of its relation to the Batalin–Fradkin–Vilkovisky (BFV) formalism, which allows for a perturbative quantisation of field theories on the possible boundary of a manifold. Despite all this, though, we will not be concerned with any quantisation in this document, so our treatment of the theory will be minimal; an exhaustive approach is found e.g. in [25], and a review of the BFV formalism is e.g. in [15]. Let us then summarise the BV formalism.

### Definition 2.1

Given a graded manifold $\mathcal {M}$, a **cohomological vector field** *Q* is an element $Q\in \mathfrak X(\mathcal {M})$ such that

2.1 $$\begin{aligned}&Q^2 = 0,  &   |Q| = 1,  &   \text{ gh }\,Q = 1, \end{aligned}$$

where one understands $Q^2$ as $Q\circ Q$, being a map $C^\infty \left( \mathcal {M}\right) \rightarrow C^\infty \left( \mathcal {M}\right)$. A manifold endowed with such a vector field is a **dg manifold**.^3^

### Remark 2.2

Given that *Q* is odd, saying that $Q^2 = 0$ is equivalent to saying that $[Q,Q] = 0$.

### Example 2.3

A paradigmatic example of dg manifold is given by the odd tangent bundle *T*[1]*M* of any non-graded manifold *M*. If $(x^i)$ are coordinates on *M* and $(\theta ^i)$ the coordinates of ghost number 1 on its fibre, then a dg structure is given by

2.2 $$\begin{aligned} Q = \theta ^i\frac{\partial }{\partial x^i}. \end{aligned}$$

### Definition 2.4

Given a vector field $X\in \mathfrak {X}(\mathcal {M})$ and a form $\omega \in \Omega (\mathcal {M})$, we say that $\omega$ is **X-invariant** if

2.3 $$\begin{aligned} \mathcal {\mathcal L }_X \omega = 0. \end{aligned}$$

### Definition 2.5

A **dg-symplectic manifold** is a graded symplectic manifold with a cohomological vector field *Q* under which the symplectic form $\omega$ is invariant. That is,

2.4 $$\begin{aligned} \mathcal {\mathcal L }_Q \omega = 0. \end{aligned}$$

### Definition 2.6

Given a graded symplectic manifold $\mathcal {M}$ and a function $f\in C^\infty \left( \mathcal {M}\right)$, the **Hamiltonian vector field** associated with *f* is the field $\mathop {f}\limits ^{\rightarrow } \in {\mathfrak X}({\mathcal {M}})$ such that

2.5 $$\begin{aligned} \mathop {f}\limits ^{\rightarrow } = (-1)^{|f| + 1}\{f,\bullet \}. \end{aligned}$$

### Definition 2.7

A **dg-Hamiltonian manifold of degree ***k* $(\mathcal {M}, H, Q, \omega )$ is a dg-symplectic manifold $\mathcal {M}$ where the symplectic form $\omega$ has ghost number *k* and the cohomological vector field *Q* is given as the Hamiltonian vector field of some degree $k+1$ function *H*, called its **Hamiltonian function**:

2.6 $$\begin{aligned} Q = (-1)^{k} \{H,\bullet \}. \end{aligned}$$

### Definition 2.8

A **classical BV theory** is a dg-Hamiltonian manifold of degree $-1$, that is, a tuple $(\mathcal F, S, Q, \omega )$ such that

1. $\mathcal F$ is a graded symplectic manifold, called the **field space**.
2. *S* is an even function over $\mathcal F$ of degree 0, called the **BV action**
3. *Q* is the cohomological, Hamiltonian vector field of *S*.
4. $\omega$ is a *Q*-invariant, odd symplectic form over $\mathcal F$ of degree $-1$.

Given that $\mathcal F$ is odd symplectic, we can locally associate each field $\phi \in \mathcal F$ to another field ${\phi ^{+}}\in \mathcal F$, known as its **antifield**, that by construction satisfies

2.7 $$\begin{aligned}&\deg _{\Omega } {\phi ^{+}}= n - \deg _{\Omega }\phi ,&\text{ gh }\, {\phi ^{+}}= -\text{ gh }\,\phi - 1. \end{aligned}$$

### Definition 2.9

A BV theory $(\mathcal F, S, Q,\omega )$ is a **BV extension** of a classical theory described by a space of fields $\mathcal {F}_{\text{ cl }}$ and an action $S_{\text{ cl }}$, if the ghost number zero part of $\mathcal F$ and of *S* correspond to $\mathcal {F}_{\text{ cl }}$ and to $S_{\text{ cl }}$, and if the restriction $\left. Q\right| _{\mathcal {F}_{\text{ cl }}}$ yields the gauge symmetries.

### Definition 2.10

Two BV theories are **weakly equivalent** if both of them are BV extensions of a same classical theory.

These theories will be **strongly equivalent** if there is a graded symplectomorphism $\Phi :\mathcal F\rightarrow \mathcal F'$ between their respective field spaces that pulls the action of one theory back to the action of the other:

2.8 $$\begin{aligned} \phi ^*S' = S. \end{aligned}$$

Such a symplectomorphism is known as a **canonical transformation**.

### Definition 2.11

Let (*q*, *p*) and $(q',p')$ be the respective even–odd coordinates of two different BV field spaces $\mathcal F$ and $\mathcal F'$. A **graded generating function of type** *j* , for $j\in [\![1,4]\!]$, is a graded function $G_j$ that we use to define two coordinates among $q,p,q',p'$ as a function of the remaining two, in one of the four following ways:

2.9a $$\begin{aligned} p&= (-1)^{|q|+1} \frac{\partial G_1(q,q')}{\partial q},&p'&= (-1)^{|q'|} \frac{\partial G_1(q,q')}{\partial q'}, \end{aligned}$$

2.9b $$\begin{aligned} p&= (-1)^{|q|+1} \frac{\partial G_2(q,p')}{\partial q},&q'&= (-1)^{|p'|} \frac{\partial G_2(q,p')}{\partial p'}, \end{aligned}$$

2.9c $$\begin{aligned} q&= (-1)^{|p|+1} \frac{\partial G_3(p,q')}{\partial p},&p'&= (-1)^{|q'|} \frac{\partial G_3(p,q')}{\partial q'}, \end{aligned}$$

2.9d $$\begin{aligned} q&= (-1)^{|p|+1} \frac{\partial G_4(p,p')}{\partial p},&q'&= (-1)^{|p'|} \frac{\partial G_4(p,p')}{\partial p'}. \end{aligned}$$

### Remark 2.12

By design, generating functions have top cohomological degree and ghost number $-1$.

### Definition 2.13

Given two graded manifolds $\mathcal {M}$, $\mathcal {N}$, and letting $\text{ Mor }(\mathcal {M}, \mathcal N)$ be the manifold of grade-preserving morphisms $\mathcal {M}\rightarrow \mathcal N$ in the category of graded manifolds, the **mapping space** $\text{ Map }(\mathcal {M}, \mathcal N)$ is the extension of $\text{ Mor }(\mathcal {M}, \mathcal N)$ that includes grade-shifting maps.

### Remark 2.14

If $\mathcal N$ is a graded vector space, then

2.10 $$\begin{aligned} \text{ Map }(\mathcal {M}, \mathcal N) = C^\infty \left( \mathcal {M}\right) \otimes \mathcal N, \end{aligned}$$

so locally the mapping space will have the form of such a tensor product of graded spaces. Details of this definition can be found in [14].

### Definition 2.15

An **AKSZ theory**^4^**in ***n* **dimensions** $(M, \mathcal N, H, Q, \alpha )$ is the combination of two things:

1. A **source** consisting of a closed and oriented *n*-manifold *M*.
2. A **target** consisting of a dg-Hamiltonian manifold $(\mathcal N, H, Q, \omega )$ of degree $n-1$ whose symplectic form $\omega$ is exact:
  2.11 $$\begin{aligned} \omega = d_\mathcal {N}\alpha \end{aligned}$$
  for $d_\mathcal {N}$ the exterior derivative on $\mathcal N$.

### Definition 2.16

Given an AKSZ theory $(M, \mathcal {N}, H_\mathcal {N}, Q_\mathcal {N}, \alpha _\mathcal {N})$ in *n* dimensions, we define the **AKSZ fields space**

2.12 $$\begin{aligned} \mathcal F = \text{ Map }(T[1] M, \mathcal N). \end{aligned}$$

Employing the notation in Remark 2.17, we take the evaluation map $\text{ ev } : T[1] M \times \mathcal F \rightarrow \mathcal N$ and define, for all $k \in \mathbb {N}$ and for coordinates $\xi$ on $\mathcal {N}$ and *X* on $\mathcal F$, its pullback as

2.13 $$\begin{aligned} \text{ ev}^* : \Omega ^k(\mathcal {N}) \rightarrow \Omega (M) \otimes \Omega ^k(\mathcal F): \beta (\xi ) \mapsto \widehat{\beta }(X), \end{aligned}$$

and we define the pushed forward projection

2.14 $$\begin{aligned} \begin{aligned} \pi _*:&\ \Omega (M) \otimes \Omega ^k(\mathcal F) \ \rightarrow \ \Omega ^k(\mathcal F), \\&\ \varphi \otimes \Phi \ \mapsto \int _M \varphi ^{\text {top}} \otimes \Phi \quad \forall \ \varphi \in \Omega (M),\ \Phi \in \Omega ^k(\mathcal F). \end{aligned} \end{aligned}$$

We construct coordinates $(X^i)$ on $\mathcal F$—the so-called **AKSZ superfields**—associated with the coordinates $(x^i)$ on $\mathcal {N}$ as

2.15 $$\begin{aligned} X^i = \text{ ev}^* x^i, \end{aligned}$$

and compose $\pi _*$ with $\text{ ev}^*$ to produce the **transgression map**

2.16 $$\begin{aligned} \mathcal T =\pi _*\text{ ev}^*. \end{aligned}$$

Letting $d_M$ be the exterior derivative on *M*, and further letting $\widetilde{d}_M, \widetilde{Q}_\mathcal {N}\in \mathfrak X(\mathcal F)$ be the respective lifts to $\mathcal F$ of $d_M$ and of $Q_\mathcal {N}$, we finally define the **AKSZ construction** associated with this theory as the tuple $(\mathcal F, S, Q, \omega )$ for

1. the **AKSZ action**
  2.17 $$\begin{aligned} S = \iota _{\widetilde{d}_M} \mathcal T \alpha _\mathcal {N}+ \mathcal T H_\mathcal {N}, \end{aligned}$$
2. the **AKSZ vector field**
  2.18 $$\begin{aligned} Q = \widetilde{d}_M + \widetilde{Q}_\mathcal {N}, \end{aligned}$$
3. and the **AKSZ symplectic form**
  2.19 $$\begin{aligned} \omega = \mathcal T\omega _\mathcal {N}. \end{aligned}$$

### Remark 2.17

Given a form $\beta \in \Omega (\mathcal {N})$, and coordinates *x* over $\mathcal {N}$ and *X* over $\mathcal F$, by $\widehat{\beta }(X)$ one understands the coordinates expression for $\beta (x)$, but symbolically replacing *x* by *X*.

Given any manifold *M* and form $\varphi \in \Omega (M)$, $\varphi ^{\text {top}}$ denotes the components of $\varphi$ with top cohomological degree, that is, those components such that $\deg _{\Omega (M)} \varphi ^{\text {top}} = \dim M$.

### Remark 2.18

The maps $\pi _*$ and $\mathcal T$ are graded:

2.20 $$\begin{aligned} |\pi _*| = |\mathcal |{T} = -\dim M. \end{aligned}$$

### Remark 2.19

By Definition 2.13, locally

2.21 $$\begin{aligned} \mathcal F \cong \Omega (M)\otimes \mathcal {N}, \end{aligned}$$

so in practice we can write *S*, *Q* and $\omega$ explicitly:

2.22 $$\begin{aligned} S&= \int _M \Bigl ( (\widehat{\alpha }_\mathcal {N})_i(X)\, d_M X^i + \widehat{H}_\mathcal {N}(X) \Bigr ), \end{aligned}$$

2.23 $$\begin{aligned} Q&= \int _M \Bigl ( d_M X^i + \widehat{Q}_\mathcal {N}^i(X) \Bigr )\ \frac{\partial }{\partial X^i}, \end{aligned}$$

2.24 $$\begin{aligned} \omega&= (-1)^{n}\int _M (\widehat{\omega }_\mathcal {N})_{ij}(X)\, d_\mathcal {N}X^i \wedge d_\mathcal {N}X^j. \end{aligned}$$

Given this, together with the fact that each superfield $X^i$ can be decomposed over summands $\{X_{(j)}^i\}_j$ of definite cohomological degree $j\in [\![0,n]\!]$, at the end of the day the AKSZ action is the action that we would obtain by

1. symbolically replacing the original fields with their associated superfields,
2. expanding those in components of definite cohomological degree,
3. keeping only the terms which have top cohomological degree.

An example of such construction is given in the next section, so let us proceed without further ado.

## 3D gravity in vacuum and *BF* theory

### Definition 3.1

An *n*-dimensional **spacetime** is a closed *n*-manifold with a **mostly positive** metric, that is, of signature $(p = n-1, n = 1)$.

### Definition 3.2

Given a principal $\text{ SO }(n-1,1)$-bundle *P* over an *n*-dimensional manifold, its **Minkowski bundle** is the associated vector bundle $(\mathcal V, \eta )$ with typical fibre $\mathbb {R}^n$, endowed with the **Minkowski metric**

3.1 $$\begin{aligned} \eta :=(-\mathbb I_{1}) \oplus \mathbb I_{(n-1)}, \end{aligned}$$

for $\mathbb I_{k}$ the identity matrix in *k* dimensions.

### Remark 3.3

Here we will focus on the case where $n=3$, so

3.2 $$\begin{aligned} \eta = \text{ diag }(-1,1,1). \end{aligned}$$

### Definition 3.4

Given a Minkowski bundle $(\mathcal V,\eta )$, a **cotriad** or **coframe field** over a 3-spacetime *M* is a non-degenerate 1-form *e* over *M* valued in $\mathcal V^{\wedge 1}$. Associated with it, there is a **triad** or **frame field** ${e^{-1}}$, which is its inverse in the following sense:

3.3 $$\begin{aligned}&e \in \Omega ^1(M, \mathcal V^{\wedge 1}),  &   {e^{-1}} \in \Omega ^1(M, \mathcal V^{\wedge 1})^*,  &   {e^{-1}}(e) = 1. \end{aligned}$$

### Remark 3.5

We talk of $\mathcal V^{\wedge 1}$ and not simply of $\mathcal V$ because we associate to the (co-)triads a multivector degree

3.4 $$\begin{aligned} \deg _\mathcal {V} e = - \deg _\mathcal {V} {e^{-1}} = 1. \end{aligned}$$

From now on, we will always conceive $\mathcal V$ as $\mathcal V^{\wedge 1}$.

### Definition 3.6

The **Palatini–Cartan formalism** in three dimensions, or simply **3D gravity**, consists of

1. an oriented spacetime *M*, assumed to have no boundary,
2. a principal $\text{ SO }(2,1)$-bundle $P \rightarrow M$, with Minkowski bundle $\mathcal V$,
3. a possibly nonzero **cosmological constant** $\Lambda \in \mathbb {R}$,

together with a field content given by

4. a cotriad $$e \in \Omega ^1(M, \mathcal V)$$ over *M*,
5. a connection 1-form $\Gamma \in \text{ Conn }(P) \cong \Omega ^1 (M, \mathcal V^{\wedge 2})$ over *P*.

With these, we define the classical field space^5^

3.5 $$\begin{aligned} \mathcal {F}_{ \text{ GR }}^{0} = \Omega ^1_{\text{ nd }}(M, \mathcal V)\times \text{ Conn }(P)^(v) \end{aligned}$$

and the classical action

3.6 $$\begin{aligned} S_{ \text{ GR }}^0(\Lambda ) = \int _M \left\langle e \wedge F_{\Gamma } + \frac{\Lambda }{6} e^{\wedge 3} \right\rangle , \end{aligned}$$

where the angle brackets $\langle \bullet \rangle$ designate the appropriate contraction of any indices other than those over $\Omega (M)$.^6^

### Remark 3.7

The reason why we can say that connections take values in $\mathcal V^{\wedge 2}$ follows from the fact that, since we work in three dimensions, $V^{\wedge 2} \cong \mathfrak {so}(2,1)$ as a Lie algebra.

### Remark 3.8

For convenience and when it is possible, we often express a particular action $S_i$ as the integral of a top form $\langle \mathscr {L}_i \rangle$:

3.7 $$\begin{aligned} S_i = \int _M \Bigl \langle \mathscr {L}_i \Bigr \rangle . \end{aligned}$$

### Remark 3.9

By *appropriate contractions*, one understands that ultimately $S_{ \text{ GR }}^0$ must be a scalar, so any internal index must be contracted. This is to say that we must correctly “trace over” the multivector indices that both *e* and $F_\Gamma$ have over $\mathcal V$, leading specifically to

3.8 $$\begin{aligned}&\bigl \langle e \wedge F_\Gamma \bigr \rangle = \epsilon _{abc}\ e^a \wedge F_\Gamma ^{bc},&\bigl \langle e^{\wedge 3} \bigr \rangle = \epsilon _{abc}\ e^a \wedge e^b \wedge e^c, \end{aligned}$$

for the 3-dimensional Levi–Civita symbol $\epsilon _{abc}$ and leaving implicit the decomposition over the generators of $\Omega (M)$.

### Definition 3.10

Given a connection 1-form $\Gamma$ and its associated covariant derivative $D_\Gamma$, the **Lie covariant derivative** $\mathcal {L}_{\xi }^\Gamma$ with respect to a vector field $\xi$ is defined as

3.9 $$\begin{aligned} \mathcal {L}_{\xi }^\Gamma = [\iota _{\xi }, D_\Gamma ]. \end{aligned}$$

### Construction 3.11

(BV extension of 3D gravity). Based on the data from 3D gravity, let $\mathcal {F}_{ \text{ GR }}$ be the field space

3.10 $$\begin{aligned} \mathcal {F}_{ \text{ GR }}= T^*[-1]\bigl ( \mathcal {F}_{ \text{ GR }}^0 \times \mathfrak X(M)[1] \times \Omega ^0(M, \text{ ad }(P)[1]) \bigr ), \end{aligned}$$

where $\mathfrak X(M)[1]$ is the space of vector fields over *M* with shifted degree and $\text{ ad }(P)[1]$ stands for the degree-shifted adjoint bundle of *P*. On the base of this shifted cotangent bundle, we denote the fields as

3.11 $$\begin{aligned} \bigl ((e, \Gamma ), \xi , \chi \bigr ) \in \mathcal {F}_{ \text{ GR }}^0 \times \mathfrak X(M)[1] \times \Omega ^0\bigl (M, \text{ ad }(P)[1]\bigr ), \end{aligned}$$

where $\xi$ encodes the diffeomorphism invariance of general relativity and $\chi$ is the ghost field required for the internal gauge transformations. In turn, the fibre includes their associated antifields, and the fact that $\mathcal {F}_{ \text{ GR }}$ is a shifted cotangent bundle allows us to define the canonical symplectic form

3.12 $$\begin{aligned} \omega _{ \text{ GR }}= \int _M \Bigl ( \delta e \delta {e^{+}} + \delta \Gamma \delta {\Gamma ^{+}}+ \delta \xi \delta {\xi ^{+}}+ \delta \chi \delta {\chi ^{+}}\Bigr ) \end{aligned}$$

Finally, we can define the action $S_{ \text{ GR }}(\Lambda ) :=S_{ \text{ GR }}^0(\Lambda ) + S_{ \text{ GR }}^1$ with

3.13 $$\begin{aligned} S_{ \text{ GR }}^1= &   \int _M \Bigl \langle {e^{+}} \bigl (\mathcal {L}_{\xi }^\Gamma e + [\chi , e]\bigr ) + {\Gamma ^{+}}\bigl (D_\Gamma \chi + \iota _{\xi }F_\Gamma \bigr ) \nonumber \\  &   + \frac{1}{2} \iota _{[\xi ,\xi ]} {\xi ^{+}}+ \frac{1}{2} {\chi ^{+}}\bigl ([\chi , \chi ] - \iota _{\xi }^2 F_\Gamma \bigr ) \Bigr \rangle , \end{aligned}$$

and its associated cohomological vector field $Q_{ \text{ GR }}$, which acts as

3.14a $$\begin{aligned} Q_{ \text{ GR }}(e)&= \mathcal {L}_{\xi }^\Gamma e + [\chi , e], \end{aligned}$$

3.14b $$\begin{aligned} Q_{ \text{ GR }}(\Gamma )&= D_\Gamma \chi + \iota _{\xi }F_\Gamma , \end{aligned}$$

3.14c $$\begin{aligned} Q_{ \text{ GR }}(\xi )&= \frac{1}{2} [\xi , \xi ] \end{aligned}$$

3.14d $$\begin{aligned} Q_{ \text{ GR }}(\chi )&= \frac{1}{2} \bigl ([\chi , \chi ] - \iota _{\xi }^2 F_\Gamma \bigr ). \end{aligned}$$

We then write $\mathscr {T}_{ \text{ GR }}:=(\mathcal {F}_{ \text{ GR }}, S_{ \text{ GR }}, Q_{ \text{ GR }}, \omega _{ \text{ GR }})$ to refer to this theory.

### Proposition 3.12

The tuple $\mathscr {T}_{ \text{ GR }}$ is a BV extension of 3D gravity, which we shall call 3-dimensional **BV gravity**.

### Definition 3.13

A *BF* **theory** in $n\ge 2$ dimensions is defined as a pair $(\mathcal F, S)$ where, given

1. an *n*-dimensional oriented manifold *M*,
2. a finite dimensional Lie group *G* and a *G*-bundle *P* over *M*,
3. a form $B\in \Omega ^{n-2}(M, \text{ ad}^*(P))$ valued in the coadjoint bundle,
4. a connection form $$A\in \text{ Conn }(P)$$,

one sets the field space to be

3.15 $$\begin{aligned} \mathcal F = \text{ ad}^*(P)\times \text{ Conn }(P) \end{aligned}$$

and the action to be

3.16 $$\begin{aligned} S = \int _M \langle B, F_A\rangle , \end{aligned}$$

where $F_A$ is the curvature of *A* and $\langle \bullet ,\bullet \rangle$ is the pairing of dual maps.

### Definition 3.14

We define *BF* **gravity** as the *BF* theory $(\mathcal {F}_{ BF}^0, S_{ BF}^0)$ where $n=3$, and where *P* is the $\text{ SO }(2,1)$-bundle over *M* with associated Minkowsky bundle $\mathcal V$ for some reference Lorentzian metric.

Moreover, for some reference connection $A'$ we interpret *B* and $A-A'$ as 1-forms valued in $\mathcal V^*$ and $\mathcal V^{\wedge 2}$, respectively, and further use the internal Minkowski metric to identify $\mathcal V^*$ with $\mathcal V$, hence seeing *B* as taking values in the latter. Thus, the field space becomes

3.17 $$\begin{aligned} \mathcal {F}_{ BF}^0 \cong \Omega ^1(M, \mathcal V) \times \Omega ^1(M,\mathcal V^{\wedge 2}). \end{aligned}$$

Finally, we incorporate a cosmological term in the action, resulting in

3.18 $$\begin{aligned} S_{ BF}^0(\Lambda ) = \int _M \bigl \langle BF_A + \frac{\Lambda }{6} B^{\wedge 3}\bigr \rangle , \end{aligned}$$

where $\langle \bullet \rangle$ denotes again the trace over the internal indices.

### Proposition 3.15

([19]). *BF* gravity is an AKSZ theory and its AKSZ extension $\mathscr {T}_{ BF}:=(\mathcal {F}_{ BF}, S_{ BF}, Q_{ BF}, \omega _{ BF})$ is strongly equivalent to 3-dimensional BV gravity. A canonical transformation $\Phi _{ BF}: S_{ \text{ GR }}\rightarrow S_{ BF}$ is provided by the following type 2 generating function:

3.19 $$\begin{aligned} G(q, p')_{\text {BF}}= &   - {B^{+}} (e - \iota _{\xi } {\Gamma ^{+}}-\frac{1}{2} \iota _{\xi }^2 {\chi ^{+}}) - A {\Gamma ^{+}}\nonumber \\  &   - {\tau ^{+}}(-\iota _{\xi }e + \frac{1}{2} \iota _{\xi }^2 {\Gamma ^{+}}- \frac{1}{3} \iota _{\xi }^3 {\chi ^{+}}) - c {\chi ^{+}}\end{aligned}$$

for $$q :=(e, {\Gamma ^{+}}, \xi , {\chi ^{+}})$$ and $$p' :=( {B^{+}}, A, {\tau ^{+}}, c)$$.

### Remark 3.16

We recall the decomposition of the fields that results from this proposition, keeping ${\tau ^{+}}$ implicit to avoid cramping the equations:

3.20a $$\begin{aligned} B&= e - \iota _{\xi } {\Gamma ^{+}}-\frac{1}{2} \iota _{\xi }^2 {\chi ^{+}},&{B^{+}}&= {e^{+}} - \iota _{\xi } {\tau ^{+}}, \end{aligned}$$

3.20b $$\begin{aligned} A&= \Gamma - \iota _{\xi } {e^{+}} + \frac{1}{2} \iota _{\xi }^2 {\tau ^{+}},&{A^{+}}&= {\Gamma ^{+}}, \end{aligned}$$

3.20c $$\begin{aligned} \tau&= - \iota _{\xi }e + \frac{1}{2} \iota _{\xi }^2 {\Gamma ^{+}}+ \frac{1}{3} \iota _{\xi }^3 {\chi ^{+}},&{\tau ^{+}}&= {e^{-1}}( {\xi ^{+}}- {e^{+}} {\Gamma ^{+}}+ \iota _{\xi } {e^{+}} {\chi ^{+}}), \end{aligned}$$

3.20d $$\begin{aligned} c&= \chi + \frac{1}{2} \iota _{\xi }^2 {e^{+}} - \frac{1}{6} \iota _{\xi }^3 {\tau ^{+}},&{c^{+}}&= {\chi ^{+}}. \end{aligned}$$

## BV supergravity

### Definition 4.1

We define **3D supergravity** and *BF* **supergravity** by extending, respectively, $\mathcal {F}_{ \text{ GR }}^0$ and $\mathcal {F}_{ BF}^0$ to

4.1 $$\begin{aligned}&{\mathcal {F}_{\text{ GR }\Phi }}^0 = \mathcal {F}_{ \text{ GR }}^0\times \Pi \mathcal {S}(P)  &   \hbox {and}  &   \mathcal {F}_{ BF\Phi }^0 = \mathcal {F}_{ BF}^0\times \Pi \mathcal {S}(P), \end{aligned}$$

for $\mathcal S(P)$ the space of 1-forms taking values in the spinor bundle associated with the principal bundle *P* and then extending their actions by a **Rarita–Schwinger action** term

4.2 $$\begin{aligned} \begin{aligned} S_{ \text{ GR }\Phi }^0(\Lambda )&= S_{ \text{ GR }}^0(\Lambda ) + \int _M \frac{1}{2} {\overline{\psi }}\, D_\Gamma \psi , \\ S_{ BF \Phi }^0(\Lambda )&= S_{ BF}^0(\Lambda ) + \int _M \frac{1}{2} {\overline{\varphi }}\, D_A \varphi . \end{aligned} \end{aligned}$$

Here the fields $\psi ,\varphi \in \Pi \mathcal S(P)$ are spin $\frac{3}{2}$ Majorana spinors.

### Remark 4.2

The connection forms will act on the spinor fields through the spin $\frac{3}{2}$ real (Majorana) representation of the algebra $\mathfrak {spin}(2,1)$. This representation will be denoted by $\boldsymbol{\rho }$ and, for the Pauli matrices $\{\sigma ^a\}_{a=1}^3$, we will use the shorthand notation $\rho ^a :=\boldsymbol{\rho }(\sigma ^a)$. Letting $\{v_a\}$ be a basis of the sections of the Minkowski bundle $\mathcal V$, we write

4.3 $$\begin{aligned} \rho :=\rho ^a v_a, \end{aligned}$$

with which the equations of motion that follow from $S_{ BF \Phi }^0$ take the following form:

4.4a $$\begin{aligned} F_A + \frac{\Lambda }{2} B \wedge B&= 0, \end{aligned}$$

4.4b $$\begin{aligned} D_A \varphi&= 0, \end{aligned}$$

4.4c $$\begin{aligned} D_A B + \frac{1}{2} {\overline{\varphi }}\, \rho \varphi&= 0. \end{aligned}$$

Meanwhile, the equations of motion issued from $S_{ \text{ GR }\Phi }^0$ are analogous, after replacing

4.5 $$\begin{aligned} (B, A, \varphi )\ \leftrightarrow \ (e, \Gamma , \psi ). \end{aligned}$$

### Proposition 4.3

*BF* supergravity is an AKSZ theory.

### Proof

We take the spacetime *M* as the source manifold, let *V* be the Minkowski space that is the typical fibre of the Minkowvski bundle $\mathcal V$, and *S*(*P*) be the vector space associated with the spinor representation $\boldsymbol{\rho }$. We hence define (*b*, *a*, *f*) as coordinates on the target

4.6 $$\begin{aligned} \mathcal {N}\cong V[1] \oplus V^{\wedge 2}[1] \oplus \Pi S(P)[1]. \end{aligned}$$

We see from this that $\mathcal {N}$ is endowed with the symplectic form

4.7 $$\begin{aligned} \omega _\mathcal {N}&= d_\mathcal {N}\alpha _\mathcal {N}, \end{aligned}$$

4.8 $$\begin{aligned} \Omega ^1(\mathcal {N})\ni \alpha _\mathcal {N}&= b\,d_\mathcal {N}a + \frac{1}{2} {\overline{f}}\, d_\mathcal {N}f, \end{aligned}$$

the Hamiltonian function

4.9 $$\begin{aligned} H_\mathcal {N}= \Bigl \langle \frac{1}{2} b[a,a] + \frac{\Lambda }{6} b^3 \Bigr \rangle + \frac{1}{2} {\overline{f}}\, a f, \end{aligned}$$

and the cohomological vector field $Q_\mathcal {N}= -\{H_\mathcal {N}, \bullet \}$ associated with $H_\mathcal {N}$ through the Poisson bracket induced by $\omega _\mathcal {N}$. $\square$

### Construction 4.4

(AKSZ extension of *BF* supergravity). Given the target established in the proof of Proposition 4.3, the field space is

4.10 $$\begin{aligned} \mathcal {F}_{ BF\Phi }\cong \Omega \bigl (M,\mathcal V\bigr )[1] \oplus \Omega \bigl (M,\mathcal V^{\wedge 2}\bigr )[1] \oplus \Omega \bigl (M, \Pi \mathcal S(P)\bigr )[1]. \end{aligned}$$

On it, the coordinates are given by the superfields

4.11a $$\begin{aligned} \widetilde{b}&= \tau + B + {A^{+}} + {c^{+}}, \end{aligned}$$

4.11b $$\begin{aligned} \widetilde{a}&= c + A + {B^{+}} + {\tau ^{+}}, \end{aligned}$$

4.11c $$\begin{aligned} \widetilde{f}&= \gamma + \varphi + {\varphi ^{+}}+ {\gamma ^{+}}, \end{aligned}$$

where summands are ordered in increasing cohomological degree, from 0 to 3, and decreasing ghost number, from 1 to $-2$. Besides, note that the parity of a field differs by 1 from the parity of its corresponding antifield. Now, replacing the classical fields in $S_{ BF \Phi }^0$ by their associated superfield, keeping only those terms of cohomological degree 3 and rearranging them, we find the BV action for *BF* supergravity:

4.12a $$\begin{aligned} S_{ BF \Phi }(\Lambda ) = \int _M \Bigl \langle \mathscr {L}_{BF \Phi }^0(\Lambda ) + \mathscr {L}_{BF \Phi }^1(\Lambda ) + \mathscr {L}_{BF \Phi }^2, \Bigr \rangle , \end{aligned}$$

where

4.12b $$\begin{aligned} \mathscr {L}_{BF \Phi }^0(\Lambda )&= BF_A + \frac{\Lambda }{6} B^{\wedge 3} + \frac{1}{2} {\overline{\varphi }}\, D_A \varphi , \end{aligned}$$

4.12c $$\begin{aligned} \mathscr {L}_{BF \Phi }^1(\Lambda )&= {B^{+}} \bigl ( [c,B] + D_A\tau \bigr ) + {A^{+}} \bigl ( D_A c + \Lambda B \tau \bigr ) \end{aligned}$$

4.12d $$\begin{aligned}&\qquad + \frac{1}{2} {c^{+}} \bigl ( [c,c] + \Lambda \tau \tau \bigr ) + {\tau ^{+}}[c,\tau ],\nonumber \\ \mathscr {L}_{BF \Phi }^2&= {\overline{\gamma }}\, {B^{+}} \varphi + \frac{1}{2} {\overline{\gamma }}\, {\tau ^{+}}\gamma + {\overline{ {\varphi ^{+}}}}\,\bigl ( D_A\gamma + c \varphi \bigr ) + {\overline{ {\gamma ^{+}}}}\, c \gamma . \end{aligned}$$

The symplectic form is given by (4.7) when we replace the coordinates by their corresponding superfields and keep only the terms of ghost number $-1$, resulting in

4.13 $$\begin{aligned} \omega _\mathcal {N}= &   \int _M \Bigl \langle d_\mathcal {N}B\; d_\mathcal {N} {B^{+}} + d_\mathcal {N}A\; d_\mathcal {N} {A^{+}} + d_\mathcal {N} {\overline{\varphi }}\; d_\mathcal {N} {\varphi ^{+}}\nonumber \\  &   + d_\mathcal {N}\tau \; d_\mathcal {N} {\tau ^{+}}+ d_\mathcal {N}c\; d_\mathcal {N} {c^{+}} + d_\mathcal {N} {\overline{\gamma }}\; d_\mathcal {N} {\gamma ^{+}}\Bigr \rangle . \end{aligned}$$

In turn, we can read off the action (4.12) the way in which the cohomological vector field acts on the coordinate fields:

4.14a $$\begin{aligned} Q_{ BF\Phi }(B)&= [c,B] + D_A\tau + {\overline{\gamma }}\, \rho \varphi ,&Q_{ BF\Phi }(\tau )&= [c,\tau ] + \frac{1}{2} {\overline{\gamma }}\, \rho \gamma , \end{aligned}$$

4.14b $$\begin{aligned} Q_{ BF\Phi }(A)&= D_A c + \Lambda B \tau ,&Q_{ BF\Phi }(c)&= \frac{1}{2} [c,c] + \frac{1}{2} \Lambda \tau \tau , \end{aligned}$$

4.14c $$\begin{aligned} Q_{ BF\Phi }(\varphi )&= D_A \gamma + c\varphi ,&Q_{ BF\Phi }(\gamma )&= c\gamma \phantom {\frac{1}{2}}. \end{aligned}$$

Thus, we conclude the construction collecting everything in a tuple

4.15 $$\begin{aligned} \mathscr {T}_{ BF\Phi }:=(\mathcal {F}_{ BF\Phi }, S_{ BF \Phi }, Q_{ BF\Phi }, \omega _{ BF\Phi }). \end{aligned}$$

### Lemma 4.5

For any degree 1 vector field $\xi \in \mathfrak X(M)[1]$, principal connection 1-form $\Gamma \in \text{ Conn }(P)$, and field $\varphi \in \Omega (M,\mathcal V)$ valued in an associated vector bundle $\mathcal V$, the following identity holds:

4.16 $$\begin{aligned} \bigl [\mathcal {L}_{\xi }^\Gamma , \iota _{\xi }\bigr ] \varphi = \iota _{[\xi ,\xi ]}\varphi . \end{aligned}$$

This is proven in [17, Lemma 9].

### Proposition 4.6

There is a ghost fermion $\varepsilon$ such that on shell

4.17 $$\begin{aligned} Q_{ \text{ GR }\Phi }(\xi )\equiv Q_{ \text{ GR }}(\xi ) + \frac{1}{2} {\overline{\varepsilon }}\, {e^{-1}}(\rho ) \varepsilon . \end{aligned}$$

### Proof

Let us call $Q':=Q_{ \text{ GR }\Phi }- Q_{ \text{ GR }}$ the on-shell extension of $Q_{ \text{ GR }}$. Now, on shell all antifields are set to zero, so (3.16) reduces to

4.18 $$\begin{aligned} (B, A, \tau , c) = (e,\Gamma , -\iota _{\xi }e, \chi ), \end{aligned}$$

which we extend additionally with $(\varphi , \gamma ) = (\psi , \kappa )$, thus being able to translate on shell the first part of (4.14a) to

4.19 $$\begin{aligned} \begin{aligned} Q_{ \text{ GR }\Phi }(e)&\equiv -D_\Gamma (\iota _{\xi }e) + [\chi , e] + {\overline{\kappa }}\, \rho \psi \\&= -\iota _{\xi }D_\Gamma e + \mathcal {L}_{\xi }^{\Gamma }e + [\chi , e] + {\overline{\kappa }}\, \rho \psi . \end{aligned} \end{aligned}$$

Meanwhile, the fact that $|e| = 2$, together with (A.5) and the definition of $\mathcal {L}_{\xi }^{\Gamma }$, implies that

4.20 $$\begin{aligned} \iota _{\xi }\bigl (\mathcal {L}_{\xi }^{\Gamma }e\bigr ) = \frac{1}{2} \bigl ([\iota _{\xi }^2, D_\Gamma ] - \iota _{[\xi ,\xi ]}\bigr ) e. \end{aligned}$$

Recalling that $Q_{ \text{ GR }}(\xi ) = \frac{1}{2} [\xi , \xi ]$, we use all this to further find that

4.21 $$\begin{aligned} \begin{aligned} Q_{ \text{ GR }\Phi }(\iota _{\xi }e)&= [Q_{ \text{ GR }\Phi }, \iota _{\xi }]e + \iota _{\xi }(Q_{ \text{ GR }\Phi }e) = \iota _{Q_{ \text{ GR }\Phi }(\xi )} e + \iota _{\xi }(Q_{ \text{ GR }\Phi }e) \\&\equiv \iota _{Q_{ \text{ GR }\Phi }(\xi )} e -\iota _{\xi }^2 D_\Gamma e + \iota _{\xi }\bigl (\mathcal {L}_{\xi }^{\Gamma }e\bigr ) + \iota _{\xi }[\chi , e] + {\overline{\kappa }}\, \rho \iota _{\xi }\psi \\&= \iota _{Q'(\xi )} e + \frac{1}{2} \iota _{[\xi , \xi ]} e - \iota _{\xi }^2 D_\Gamma e + \frac{1}{2} \iota _{\xi }^2 D_\Gamma e - \frac{1}{2} D_\Gamma (\iota _{\xi }^2 e) \\&\qquad - \frac{1}{2} \iota _{[\xi ,\xi ]} e + \iota _{\xi }[\chi ,e] + {\overline{\kappa }} \, \rho \iota _{\xi }\psi \\&= \iota _{Q'(\xi )} e -\frac{1}{2} \iota _{\xi }^2 D_\Gamma e + \iota _{\xi }[\chi , e] + {\overline{\kappa }}\, \rho \iota _{\xi }\psi .\\ \end{aligned} \end{aligned}$$

Moreover, after adapting to on-shell 3D supergravity both the equation of motion (4.4c) and the second part of (4.14a), from what precedes we deduce that

4.22 $$\begin{aligned} \begin{aligned} Q_{ \text{ GR }\Phi }(\iota _{\xi }e)&\equiv \iota _{Q'(\xi )} e + \frac{1}{2} \iota _{\xi } {\overline{\psi }}\, \rho \iota _{\xi }\psi + \iota _{\xi }[\chi , e] + {\overline{\kappa }}\, \rho \iota _{\xi }\psi \\&\equiv -Q_{ \text{ GR }\Phi }(\tau ) \equiv \iota _{\xi }[\chi , e] -\frac{1}{2} {\overline{\kappa }}\, \rho \kappa , \end{aligned} \end{aligned}$$

which holds if and only if

4.23 $$\begin{aligned} \iota _{Q'(\xi )} e \equiv -\frac{1}{2} ( {\overline{\kappa }}+ \iota _{\xi } {\overline{\psi }})\, \rho (\kappa + \iota _{\xi }\psi ). \end{aligned}$$

With this, we finally conclude that

4.24 $$\begin{aligned} \begin{aligned} Q_{ \text{ GR }\Phi }(\xi )&= Q_{ \text{ GR }}(\xi ) + {e^{-1}}\bigl (e(Q'\xi )\bigr ) = Q_{ \text{ GR }}(\xi ) - {e^{-1}}\bigl (\iota _{Q' (\xi )} e\bigr ) \\&\equiv Q_{ \text{ GR }}(\xi ) + \frac{1}{2} {\overline{\varepsilon }}\, {e^{-1}}(\rho ) \varepsilon \end{aligned} \end{aligned}$$

for the ghost Majorana fermion $\varepsilon :=\kappa + \iota _{\xi }\psi$. $\square$

### Remark 4.7

This property is expected, since the generators of supersymmetry square to translation generators, and the former are to be encoded by ghost fermions while the latter are realised through $\xi$.

### Remark 4.8

Our current goal being to extend (3.19) as to find a BV theory of 3D supergravity that encodes explicitly both supersymmetry and diffeomorphism invariance, this last proposition will serve us as guiding principle. Indeed, we will be searching for a type 2 generating function $G_{\text {BF}\Phi }$ that decomposes as

4.25 $$\begin{aligned} G_{\text {BF}\Phi } = G_{\text {BF}} + G_{ BF}^{\text{ ext }}, \end{aligned}$$

and evidently we would like $G^{\text{ ext }}_{\text{ BF }}$ to be a minimal extension, that is, as simple as possible without being ineffective. This *without being ineffective* is precisely what Proposition 4.6 addresses: we must ensure that the extended symplectomorphism $\Phi _{ BF\Phi }: {\mathcal {F}_{\text{ GR }\Phi }}\rightarrow \mathcal {F}_{ BF\Phi }$ leads to a cohomological vector field that on shell is equal to (4.17). Fortunately, finding such extension is eased by the next proposition.

### Proposition 4.9

A minimal extension $G_{ BF}^{\text{ ext }}$ of $G_{ BF}$ ensuring that equation (4.17) holds on shell can only depend on spinorial fields or on contractions of those with respect to $\xi$.

### Proof

As before, we denote by $(\varphi , \gamma )$ the spinorial field and ghost in $\mathcal {F}_{ BF\Phi }$ and by $(\psi , \varepsilon )$ the corresponding pair on ${\mathcal {F}_{\text{ GR }\Phi }}$. Since the extension we are looking for aims at being minimal, all its terms must be spinorial scalars, because if any term in $G_{ BF}^{\text{ ext }}$ did not include spinors, it would effectively amount to a modification of $G_{ BF}$ spoiling the known canonical transformation between 3D and *BF* gravities in the absence of fermions. Consequently, all terms in $G_{ BF}^{\text{ ext }}$ should take the form

4.26 $$\begin{aligned} {\overline{y}} x y' \end{aligned}$$

where *y* and $y'$ are spinors in $\mathcal {F}_{ BF\Phi }$ and in ${\mathcal {F}_{\text{ GR }\Phi }}$ respectively, and *x* is any product of non-spinorial fields—possibly including contractions—in either theory. Of course, not any such combination is valid, given that every such product must have cohomological degree 3 and ghost number $-1$, and every internal index must be contracted. In fact, under these constraints there will be at most two kinds of valid products ${\overline{y}} x y'$. The first kind will have $x=1$ and an appropriate distribution of contractions $\iota _{\xi }$, only consisting—up to redistribution of the $\iota _{\xi }$—of the following possible pairs $(y,y')$:

4.27 $$\begin{aligned} \begin{aligned}&( {\gamma ^{+}}, \varepsilon ),  &   ( {\varphi ^{+}}, \psi ),  &   ( {\gamma ^{+}}, \iota _{\xi }\psi ), \\&(\iota _{\xi } {\varphi ^{+}}, {\psi ^{+}}),  &   (\iota _{\xi }^2 {\varphi ^{+}}, {\varepsilon ^{+}}),  &   (\iota _{\xi }^3 {\gamma ^{+}}, {\varepsilon ^{+}}), \end{aligned} \end{aligned}$$

or the analogous pairings exchanging the roles of the fields in ${\mathcal {F}_{\text{ GR }\Phi }}$ by those in $\mathcal {F}_{ BF\Phi }$ and vice versa. Meanwhile, the second kind of product will have $x\ne 1$, yet it is evident that any product of this type, to be valid, should be obtained from a product of the first kind by replacing any number of contractions with a product *x* of non-spinorial fields that have the same cohomological degree and ghost number as the power of $\iota _{\xi }$ that they are replacing. In other words,

4.28 $$\begin{aligned} \begin{pmatrix} \deg _{\Omega }x \\ \text{ gh }\,\;x \end{pmatrix} = \begin{pmatrix} -k \\ k \end{pmatrix} \end{aligned}$$

for some $k\in \mathbb {N}$. Now, every non-spinorial field—including the contraction $\iota _{\xi }$—has its pair $(\deg _{\Omega }\bullet , \text{ gh }\,\bullet )$ among the following:

4.29 $$\begin{aligned} v_1 = \begin{pmatrix} 0 \\ 1 \end{pmatrix},\ v_2 = \begin{pmatrix} 1 \\ 0 \end{pmatrix},\ v_3 = \begin{pmatrix} 2 \\ -1 \end{pmatrix},\ v_4 = \begin{pmatrix} 3 \\ -2 \end{pmatrix},\ v_5 = \begin{pmatrix} -1 \\ 1 \end{pmatrix}, \end{aligned}$$

which, respectively, correspond to the degree pairs of *c*, *A*, $$ {B^{+}}$$, $$ {c^{+}}$$ and $$\iota _{\xi }$$, in that order. The question, then, reduces to solving the simple equation

4.30 $$\begin{aligned} \begin{aligned} k^i v_i = 0  &   \text {for}  &   \{k^i\}_{i=1}^4\subset \mathbb {N},\ k^5\in \mathbb Z, \end{aligned} \end{aligned}$$

which holds if and only if $k^i=0$ for all *i*. This is equivalent to saying that any valid product ${\overline{y}} x y$ is of the first kind, that is, a Dirac product of spinors or of contractions of those with respect to $\xi$. $\square$

### Remark 4.10

Proposition 4.9 facilitates the labour notably by making $G_{ BF}^{\text{ ext }}$ include at most four terms, which, moreover, will show a convenient property: they will only fix the spinorial fields and, at most, modify the expression for ${\xi ^{+}}$ as a function of the fields in $\mathcal {F}_{ BF\Phi }$. Finding an appropriate extension, then, is letting

4.31 $$\begin{aligned} G_{ BF}^{\text{ ext }}= \sum _i k_i {\overline{y_i}} y'_i \end{aligned}$$

and determining the (at most) four parameters $k_i$ that will lead to a $Q_{ \text{ GR }\Phi }$ that on shell satisfies (4.17) and to an action $S_{ \text{ GR }\Phi }$ whose classical spinorial part is $\frac{1}{2} \psi \, D_\Gamma \psi$.

### Theorem 4.11

A BV extension of 3D supergravity is provided by the tuple $\mathscr {T}_{ \text{ GR }\Phi }:=({\mathcal {F}_{\text{ GR }\Phi }}, S_{ \text{ GR }\Phi }, Q_{ \text{ GR }\Phi }, \omega _{{ GR}\Phi })$ for

4.32a $$\begin{aligned} {\mathcal {F}_{\text{ GR }\Phi }}&= \mathcal {F}_{ \text{ GR }}\times T^*[-1]\Omega (M, \Pi \mathcal S(P)), \end{aligned}$$

4.32b $$\begin{aligned} S_{ \text{ GR }\Phi }&= {\Phi _{ BF\Phi }}^*S_{ BF \Phi }, \end{aligned}$$

4.32c $$\begin{aligned} \omega _{{ GR}\Phi }&= {\Phi _{ BF\Phi }}^*\omega _{ BF\Phi }, \end{aligned}$$

4.32d $$\begin{aligned} Q_{ \text{ GR }\Phi }&= \{\bullet , S_{ \text{ GR }\Phi }\}, \end{aligned}$$

where the Poisson bracket is defined by $\omega _{{ GR}\Phi }$ and the canonical transformation $\Phi _{ BF\Phi }$ is generated by

4.33 $$\begin{aligned} G_{ BF\Phi }= G_{ BF}+ G_{ BF}^{\text{ ext }}, \end{aligned}$$

where $G_{ BF}$ is the generating function (3.19) and $G_{ BF}^{\text{ ext }}$ is given as

4.34 $$\begin{aligned} G_{ BF}^{\text{ ext }}(q,p') = - {\overline{ {\varphi ^{+}}}}\, \psi - {\overline{ {\gamma ^{+}}}}\, (\varepsilon - \iota _{\xi }\psi ) \end{aligned}$$

for $$q:=(e, {\Gamma ^{+}}, \xi , {\chi ^{+}}, \psi , \varepsilon )$$ and $$p':=( {B^{+}}, A, {\tau ^{+}}, c, {\varphi ^{+}}, {\gamma ^{+}})$$.

### Proof

This generating function leads to

4.35a $$\begin{aligned}&\varphi = \psi ,  &   {\varphi ^{+}}= {\psi ^{+}}- \iota _{\xi } {\varepsilon ^{+}}, \end{aligned}$$

4.35b $$\begin{aligned}&\gamma = \varepsilon - \iota _{\xi }\psi ,  &   {\gamma ^{+}}= {\varepsilon ^{+}}, \end{aligned}$$

so following Remark 4.10, we only have to attend some of the terms in $S_{ BF \Phi }$ (4.12) to check whether it produces an extension of classical 3D supergravity. Firstly, since Definition (3.20b) of *A* in terms of fields in ${\mathcal {F}_{\text{ GR }\Phi }}$ remains unchanged, the expansion of the classical spinorial field gives

4.36 $$\begin{aligned} \frac{1}{2} {\overline{\varphi }}\, D_A \varphi = \frac{1}{2} {\overline{\psi }}\, D_\Gamma \psi - \frac{1}{2} {\overline{\psi }}\, (\iota _{\xi } {e^{+}} - \frac{1}{2} \iota _{\xi }^2 {\tau ^{+}}) \psi , \end{aligned}$$

so indeed the classical spinorial term is recovered on shell—where, remember, antifields are set to zero. Secondly, since the only terms modifying $Q_{ \text{ GR }}(\xi )$ are those spinorial terms in $S_{ BF \Phi }$ that include a factor of ${\tau ^{+}}$—because only these depend on ${\xi ^{+}}$, specifically through ${e^{-1}}( {\xi ^{+}})$—we only verify the following terms in $\mathscr {L}_{BF \Phi }$:

4.37 $$\begin{aligned} \frac{1}{2} {\overline{\psi }}\, A \psi + {\overline{\gamma }}\, {B^{+}} \varphi + \frac{1}{2} {\overline{\gamma }}\, {\tau ^{+}}\gamma + {\overline{ {\varphi ^{+}}}}\, A \gamma + {\overline{ {\varphi ^{+}}}}\, c \varphi + {\overline{ {\gamma ^{+}}}}\, c \gamma . \end{aligned}$$

After expansion—which is rendered explicit in Definition 4.13—one verifies that

4.38 $$\begin{aligned} Q_{ \text{ GR }\Phi }(\xi ) = \frac{1}{2} [\xi , \xi ] + \frac{1}{2} {\overline{\varepsilon }}\, {e^{-1}} (\rho ) \varepsilon + \cdots \end{aligned}$$

omitting all terms that contain antifields, so indeed $Q_{ \text{ GR }\Phi }$ satisfies (4.17). Finally, (4.34) holds necessarily, since (4.32a) merely accounts for the incorporation of fermions, while equations (4.32b) to (4.32d) follow from the definition of a canonical transformation and the fact that $G_{ BF\Phi }$ is a generating function. Therefore, we have constructed a theory $\mathscr {T}_{ \text{ GR }\Phi }$ that is symplectomorphic to *BF* supergravity, and moreover, $\mathscr {T}_{ \text{ GR }\Phi }$ produces classical 3D supergravity on shell; in other words, $\mathscr {T}_{ \text{ GR }\Phi }$ is a BV extension of 3D supergravity. $\square$

### Remark 4.12

Due to the fact that it only involves spinors and their contraction, the only equation in (3.16) that the extension $G_{ BF}^{\text{ ext }}$ modifies is the one corresponding to ${\tau ^{+}}$, giving

4.39 $$\begin{aligned} {\tau ^{+}}= {e^{-1}} \bigl ( {\xi ^{+}}- {e^{+}} {\Gamma ^{+}}- {\overline{ {\gamma ^{+}}}} \psi + \iota _{\xi } {e^{+}} {\chi ^{+}}\bigr ). \end{aligned}$$

Having established this result, we find ourselves in a position to give an explicit form for 3D supergravity.

### Definition 4.13

We will call 3-dimensional **BV supergravity** the theory built in Theorem 4.11. Its action is given by

4.40a $$\begin{aligned} S_{ \text{ GR }\Phi }= S_{ \text{ GR }}+ \int _M \Bigl \langle \mathscr {L}_{\text{ GR }\Phi }^1 \Bigr \rangle , \end{aligned}$$

for the density

4.40b $$\begin{aligned} \mathscr {L}_{\text{ GR }\Phi }^1 = \frac{1}{2} {\overline{\psi }}\, D_\Gamma \psi + {\overline{\psi }}\, {e^{+}} \varepsilon + \frac{1}{2} {\overline{\varepsilon }}\, {\tau ^{+}}\varepsilon + {\overline{ {\psi ^{+}}}} \, Q_{ \text{ GR }\Phi }(\psi ) + {\overline{ {\varepsilon ^{+}}}} \, Q_{ \text{ GR }\Phi }(\varepsilon ), \end{aligned}$$

where ${\tau ^{+}}$—as given in (4.39)—is kept implicit for the sake of readability. In turn, the cohomological vector field decomposes as

4.41a $$\begin{aligned} Q_{ \text{ GR }\Phi }= Q_{ \text{ GR }}+ Q^{\text{ ext }}_{\text{ GR }}, \end{aligned}$$

for an extension that acts in the following manner:

4.41b $$\begin{aligned}&Q^{\text{ ext }}_{\text{ GR }}(\Gamma ) = Q^{\text{ ext }}_{\text{ GR }}(\chi ) = 0, \end{aligned}$$

4.41c $$\begin{aligned}&Q^{\text{ ext }}_{\text{ GR }}(e) = {\overline{\psi }}\, \rho \varepsilon - \iota _{\xi } {\overline{ {\psi ^{+}}}} \, \rho \kappa - \frac{1}{2} \iota _{\xi }^2 {\overline{ {\psi ^{+}}}} \, \rho \psi \end{aligned}$$

4.41d

4.41e $$\begin{aligned}&Q_{ \text{ GR }\Phi }(\psi ) = \chi \psi + D_\Gamma \kappa - \iota _{\xi } {e^{+}} \kappa + \frac{1}{2} \iota _{\xi }^2 {e^{+}} \psi + \frac{1}{2} \iota _{\xi }^2 {\tau ^{+}}\kappa - \frac{1}{6} \iota _{\xi }^3 {\tau ^{+}}\psi , \end{aligned}$$

4.41f $$\begin{aligned}&Q_{ \text{ GR }\Phi }(\varepsilon ) = \chi \varepsilon + \iota _{\xi }D_\Gamma \kappa - \frac{1}{2} \iota _{\xi }^2 {e^{+}} (\varepsilon - 2 \iota _{\xi }\psi ) + \frac{1}{6} \iota _{\xi }^3 {\tau ^{+}}(2\varepsilon - 3 \iota _{\xi }\psi ), \end{aligned}$$

writing $$\kappa :=(\varepsilon - \iota _{\xi }\psi )$$ and $$\underline{\rho }:={e^{-1}} (\rho )$$.

## Data Availability

The manuscript has no associated data.

## Appendix A Selected results in graded geometry

### Proposition A.1

As their non-graded counterparts, graded Lie derivatives satisfy

A.1 $$\begin{aligned} [\mathcal {\mathcal L }_X, \mathcal {\mathcal L }_Y] = \mathcal {\mathcal L }_{[X,Y]}\quad \forall \ X,Y\in \mathfrak X(\mathcal {M}). \end{aligned}$$

### Remark A.2

This is a direct consequence of their definition.

### Proposition A.3

**Cartan’s identity**—or Cartan’s **magic formula**—relates the graded interior derivative, the exterior derivative, and the graded Lie derivative:

A.2 $$\begin{aligned} [\iota _X, d] = \mathcal {\mathcal L }_X. \end{aligned}$$

### Remark A.4

The proof is verbatim the one used in conventional differential geometry, but keeping track of the gradings.

### Proposition A.5

For any graded vector fields $X,Y\in \mathfrak X(\mathcal {M})$ and form $\omega \in \Omega (\mathcal {M})$ over a graded manifold $\mathcal {M}$, we have

A.3a $$\begin{aligned} [\mathcal {\mathcal L }_X, \iota _Y]\omega = \iota _{[X,Y]}\omega \end{aligned}$$

### Proof

The behaviour of the interior and Lie derivatives, as for any derivation, will be fully determined by their action on an arbitrary function $f \in C^\infty \left( \mathcal {M}\right)$ and on its differential *df*. Now, on the one hand it is obvious that

A.4 $$\begin{aligned} [\mathcal {\mathcal L }_X, \iota _Y]f = \iota _{[X,Y]}f = 0. \end{aligned}$$

On the other hand, recalling that $|\iota _Y| = |Y| - 1$, Cartan’s identity and $d^2 = 0$ together imply that

A.5a $$\begin{aligned} \begin{aligned} \iota _Y (L{\mathcal L_{X}} df)&= \iota _Y ([\iota _X, d] df) = -(-1)^{|\iota _X|} \iota _Y d(Xf) \\&= (-1)^{|X|} Y(X f). \end{aligned} \end{aligned}$$

This, jointly with $|L{\mathcal L_{X}}| =|X|$, lets us conclude that

A.5b $$\begin{aligned} \begin{aligned} {[}L{\mathcal L_{X}}, \iota _Y{]} df&= L{\mathcal L_{X}} (\iota _Y df) - (-1)^{|L{\mathcal L_{X}}| |\iota _Y|} \iota _Y (L{\mathcal L_{X}} df) \\&= X (Y f) - (-1)^{|X| (|Y| - 1)}(-1)^{|X|} Y (X f) = [X, Y] f \\&= \iota _{[X, Y]} df, \end{aligned} \end{aligned}$$

thus proving the proposition. $\square$

## Footnotes

1 A general review of the Palatini formalism is found in [27], one of the PCH formalisms in [16], and Holst’s original work is [23].

2 For an introduction to supersymmetry, see [21], and see [26] for an introduction to supergravity, while the reader is referred to [30] for a thorough exposition of both.

3 Here *dg* stands for *differential graded*.

4 Here we focus on this special case of the more general AKSZ method.

5 where “nd” stands for *non-degenerate*.

6 The expression within the brackets actually defines a density, which could be integrated over a non-orientable manifold, yet for simplicity we assume orientability.
